# Machine learning based development of an early diagnosis signature for distinguishing hospitalized pediatric human respiratory syncytial virus infection from mycoplasma pneumonia

**DOI:** 10.3389/fped.2026.1845227

**Published:** 2026-06-02

**Authors:** Xiandan Chen, Linlu Ying, Weixing Kong, Wangxiong Hu, Zhong Hu

**Affiliations:** 1 Department of Pediatrics, Yongkang Women and Children's Health Hospital, Yongkang, Zhejiang, China; 2 Department of Clinical Medicine, Xiantao Vocational College, Xiantao, Hubei, China; 3 Cancer Institute, Key Laboratory of Cancer Prevention and Intervention, Ministry of Education, the Second Affiliated Hospital, Zhejiang University School of Medicine, Hangzhou, Zhejiang,,China

**Keywords:** blood signature, community-acquired pneumonia, human respiratory syncytial virus, lactic dehydrogenase, LASSO, mycoplasma pneumoniae

## Abstract

**Background:**

The differentiation between human respiratory syncytial virus (HRSV) and mycoplasma pneumoniae (MP) infections in pediatric community-acquired pneumonia (CAP) remains a clinical challenge due to overlapping respiratory symptoms. A rapid, non-invasive diagnostic tool is urgently needed to guide appropriate therapeutic decisions and antimicrobial stewardship.

**Objective:**

This study aimed to develop and validate a blood-based biomarker signature for distinguishing HRSV from MP infections.

**Methods:**

We conducted a retrospective cohort study analyzing clinical data and blood samples from patients with CAP infected by HRSV and MP, diagnosed via PCR and serology. Patients were randomly split into a discovery cohort and a validation cohort. Least Absolute Shrinkage and Selection Operator (LASSO) regression was used for feature selection. ML was employed to train, optimize, and evaluate multiple classifiers, including logistic regression, random forest, and support vector machines. The diagnostic performance of the final model was evaluated by the area under the receiver operating characteristic curve (AUC), sensitivity, and specificity.

**Results:**

The analysis identified a parsimonious five-biomarker signature comprising eosinophilic granulocyte, immunoglobulin A, lactic dehydrogenase (LDH), β2-microglobulin, and the albumin to globulinratio (AGR). The optimized random forest model demonstrated superior performance, achieving an AUC-ROC of 0.89 (95% CI: 0.85–0.90) for distinguishing HRSV from MP.

**Conclusion:**

We developed and validated a novel, minimally invasive blood biomarker signature that accurately distinguishes HRSV from MP infections. This model has the potential to serve as a valuable adjunctive tool for early etiological diagnosis, facilitating timely and targeted clinical management. Further prospective, multi-center studies are warranted to confirm its generalizability and clinical utility.

## Background

Respiratory tract infections represent a significant cause of morbidity and mortality in the pediatric population globally (1). Among the diverse etiological agents, *Mycoplasma pneumoniae* (MP) and human respiratory syncytial virus (HRSV) stand out as two of the most prevalent and clinically impactful pathogens, particularly in the context of community-acquired pneumonia (CAP) and lower respiratory tract infections (LRTIs) (2). Both pathogens predominantly affect young children, leading to substantial healthcare burdens through hospitalizations, intensive care unit admissions, and associated economic costs (3, 4). The clinical presentations of MP pneumonia (MPP) and HRSV-associated LRTIs, such as bronchiolitis and pneumonia, often exhibit overlapping features including fever, cough, tachypnea, and wheezing, making their differentiation based solely on clinical grounds a formidable challenge for clinicians (5, 6). This diagnostic ambiguity is further compounded by the nonspecific nature of initial symptoms and the common practice of empirical antibiotic therapy, which is ineffective against viral HRSV infections (7). Consequently, the inability to rapidly and accurately distinguish between these two etiologies can lead to inappropriate antimicrobial use, delayed initiation of appropriate supportive care, and suboptimal patient outcomes, highlighting an urgent need for reliable diagnostic tools.

Current diagnostic modalities for MP and HRSV primarily rely on nucleic acid amplification test from nasopharyngeal or throat swabs, which offer high sensitivity and specificity for direct pathogen detection (8). However, these tests have inherent limitations. They require specialized equipment and trained personnel, may not be readily available in all clinical settings, particularly in primary care or resource-limited regions, and their results are not instantaneous. Furthermore, a positive polymerase chain reaction (PCR) result for one pathogen does not definitively rule out a concurrent infection or indicate the dominant driver of the current severe clinical phenotype, especially in cases of viral-bacterial co-detection. Serological tests for MP, detecting IgM antibodies, are hampered by a delay in antibody response and potential cross-reactivity, limiting their utility in acute clinical decision-making. Therefore, while molecular diagnostics are the gold standard, there is a clear gap for a rapid, host-centric biomarker signature that can reflect the real-time pathophysiological state and aid in early etiological differentiation.

The host immune and inflammatory responses to MP and HRSV infections, while sharing some common pathways, exhibit distinct characteristics that form the biological rationale for a differential biomarker signature. Numerous studies have consistently identified C-reactive protein (CRP), lactate dehydrogenase (LDH), and the neutrophil-to-lymphocyte ratio (NLR) as key indicators of disease severity and progression in pediatric MPP (9). Elevated levels of interleukin-6 (IL-6), IL-10, interferon-gamma (IFN-*γ*), D-dimer, and ferritin have also been strongly associated with refractory or severe courses, suggesting a role for cytokine storm, hypercoagulability, and immune dysregulation (10, 11).

In contrast, the host response to HRSV is shaped by its nature as a respiratory virus with specific immunomodulatory strategies. A critical feature of HRSV infection is the impaired and age-dependent production of Immunoglobulin A (IgA) at the respiratory mucosa, which is crucial for neutralizing viral replication; infants fail to mount robust IgA responses compared to adults, contributing to disease severity (12). While systemic inflammatory markers like CRP may also rise in severe HRSV infections, the specific cytokine and chemokine profile, such as the role of type I and III interferons and specific mediators like IL-6 and CXCL8 in the airway epithelium, may differ from that triggered by MP (13). The distinct Th2-biased immune responses caused by HRSV also point to unique immunological pathways that could be reflected in host biomarkers (14, 15).

However, few signatures have been specifically validated for discriminating between two common and clinically overlapping pathogens like HRSV and MP. A robust, blood-based classifier could transform clinical practice by providing an objective measure to withhold unnecessary antibiotics in HRSV cases while ensuring their prompt administration for MP pneumonia.

Therefore, we designed this study to develop and validate a blood biomarker signature for predicting HRSV vs. MP infection in patients presenting with CAP. We hypothesize that a multi-parameter model derived from host immune markers in peripheral blood will accurately discriminate between these two pathogens, offering a rapid adjunct to current diagnostic methods. Future research must focus on prospective, multicenter studies to discover, refine, and validate such a signature across diverse populations and epidemiological settings.

## Methods

### Study design and population

This retrospective cohort study was conducted at Yongkang Maternal and Child Health Hospital (YMCHH) between 2023 and 2024. We included pediatric patients admitted with a clinical and radiographic diagnosis of pneumonia. Patients with incomplete medical records, pre-existing neuromuscular disorders, or recent trauma/surgery were excluded. Inclusion criteria: 1. Hospitalized pediatric patients (aged ≤12 years) with a clinical diagnosis of acute LRTI, such as bronchiolitis or pneumonia. 2. Availability of a paired nasopharyngeal swab or aspirate sample tested via multiplex PCR or reverse transcription PCR (RT-PCR) to confirm infection with either HRSV or MP as the identified pathogen. 3. Availability of a corresponding residual serum or plasma sample, collected within 48 h of admission prior to any immunotherapy (e.g., intravenous immunoglobulin). 4. Availability of a complete medical record, including demographic data, clinical presentation, and standard laboratory results (e.g., complete blood count, CRP). Exclusion criteria: 1. Patients with pre-existing neuromuscular disorders (e.g., muscular dystrophy and myopathies) or infected by other pathogen (e.g., SARS-CoV-2, haemophilus influenza, rhinovirus, human metapneumovirus, human adenovirus, and influenza A/B virus). 2. Presence of a known chronic medical condition that could significantly alter immune or inflammatory responses, including but not limited to: primary or secondary immunodeficiency, active malignancy, congenital heart disease, chronic lung disease (e.g., cystic fibrosis, bronchopulmonary dysplasia), or autoimmune disorders. 3. Recent (within four weeks prior to admission) administration of systemic corticosteroids, immunomodulators, or macrolide antibiotics (for the MP cohort). 4. Missing or incomplete laboratory data. 5. Hospital-acquired pneumonia cases or those with prior antibiotic use within 48 h before admission. The research protocol was approved by the Medical Ethics Committee of the YMCHH.

### Data collection and definitions

Demographic data (age, sex), clinical characteristics (symptom duration, comorbidities), radiographic findings (chest x-ray or CT) and clinical outcomes (length of hospital stay, days of fever and cough duration, and mortality), and laboratory results at initial presentation were extracted from the electronic medical record (EMR). The primary outcome was the confirmed etiological diagnosis of HRSV infection (HRSV-positive group) or MP infection (MP-positive group), serving as the binary classification target for model development.

### Blood biomarker measurement

For all included patients, residual serum samples collected at initial presentation (prior to specific treatment) were retrieved from the hospital's central biobank. A panel of 15 candidate blood-based biomarkers was quantified using standardized, commercially available assays. The panel was selected based on their established or putative roles in host immune response, inflammation, and tissue damage in respiratory infections. It included: serum creatine kinase (CK) levels (U/L), creatine kinase isoenzyme MB (U/L), immunoglobulin M/G/A (g/L), LDH (U/L), CRP (mg/L), globulin (g/L), D-dimer (mg/L), *β*2-microglobulin(g/L), white blood cell (WBC) count (×10^9^/L), the albumin to globulin ratio (AGR, %), eosinophilic granulocyte (%), procalcitonin (ng/mL), and glutamic-pyruvic transaminase (U/L). All assays were performed in batch by laboratory personnel blinded to the clinical diagnosis, following manufacturers' protocols. Internal quality controls were used for each assay run. Missing data, which constituted less than 10% of the dataset, were imputed using multivariate imputation by chained equations (MICE) (16).

### Statistical analysis

Data analysis was performed using R software (version 4.3.1). Continuous variables were reported as median (interquartile range, IQR) and compared using the Mann–Whitney *U*-test. Categorical variables were reported as frequencies and compared using Chi-square or Fisher's exact test. A two-tailed *P*-value < 0.05 was considered statistically significant.

### Feature selection

The dataset was partitioned into a training set (70% of patients) and a hold-out internal validation set (30%). To identify the most relevant predictors and mitigate overfitting, a two-stage feature selection approach was employed. First stage: the conventional statistical analysis identified biomarkers with significant differential levels (*p* < 0.05) between HRSV and MP groups. Then, to avoid overfitting and collinearity, the Least Absolute Shrinkage and Selection Operator (LASSO) regression with 10-fold cross-validation was applied to these significant biomarkers. This method shrinks coefficients of less contributory variables to zero, selecting a parsimonious set of the most informative predictors for the final signature.

### Model training and tuning

The features selected by the LASSO was subjected to multiple algorithms including LASSO, linear discriminant analysis (LDA), extreme gradient boosting (Xgboost), logistic regression (Logistic), random forest (RF), KK-nearest neighbors (KKNN), support vector machine (SVM), decision tree (DT) and naïve bayes (NB) for model development. The efficacy of the eight ML algorithms was performed using the *mlr3* learner package (17). The comparative effectiveness of these models was measured by the area under the receiver operating characteristic curve (AUC) score to identify the best model. After identifying the optimal model, its performance on the validation dataset was assessed in terms of sensitivity and specificity. Model hyperparameters were tuned via 10-fold cross-validation on the training set using the *mlr3tuning* package with a grid search strategy.

## Results

### Demographic characteristics and clinical information

In this study, a total of 1,339 pediatric patients (476 HRSV and 863 MP) diagnosed with pneumonia were initially included in the analysis. Among them, 276 patients were excluded for further analysis because missing laboratory data, presence of a known chronic medical condition, or coinfected by other pathogens (Supplementary Figure S1). Of the remaining 1,063 children hospitalized for pneumonia, they were aged one months to 12 years (median age: 3 years); 58% were male. Notably, CRP (4 vs. 2, *P* < 0.001), eosinophilic granulocyte (0.12 vs. 0.07, *P* < 0.001), immunoglobulin M (1.19 vs. 0.94, *P* < 0.001), immunoglobulin G (9.8 vs. 7.0, *P* < 0.001), IgA (1.2 vs. 0.44, *P* < 0.001), and globulin (28.3 vs. 24.2, *P* < 0.001) in the MP group were higher than those in the HRSV group. In contrast, AGR (1.8 vs. 1.5, *P* < 0.001), procalcitonin (0.10 vs. 0.07, *P* < 0.001), glutamic-pyruvic transaminase (17 vs. 13, *P* < 0.001), creatine kinase (CK, 106 vs. 94, *P* < 0.001), creatine kinase isoenzyme MB (32 vs. 24, *P* < 0.001), LDH (356 vs. 307, *P* < 0.001), and β2-microglobulin (2.79 vs. 2.06, *P* < 0.001) in the HRSV group were higher than those in the MP group. There were no significant differences in age, D-dimer, WBC count, coughing days, fevering days, and inpatient days between the two groups (Table 1).

**Table 1:** The clinical and laboratory characteristics of the pediatric pneumonia caused by HRSV and MP.

Characteristics	HRSV	MP	*p*-value^b^
	*N* = 341^a^	*N* = 722^a^	
CRP	2 (1, 6)	4 (1, 11)	<0.001
Eosinophilic granulocyte	0.07 (0.02, 0.16)	0.12 (0.04, 0.23)	<0.001
White blood cell count	7.6 (6.0, 10.1)	7.8 (6.1, 9.8)	0.5
Procalcitonin	0.10 (0.06, 0.20)	0.07 (0.05, 0.13)	<0.001
Glutamic-pyruvic transaminase	17 (14, 23)	13 (11, 16)	<0.001
Creatine kinase	106 (78, 149)	94 (71, 126)	<0.001
Creatine kinase isoenzyme MB	32 (25, 42)	24 (19, 35)	<0.001
IgM	0.94 (0.69, 1.23)	1.19 (0.92, 1.49)	<0.001
IgG	7.0 (5.3, 8.7)	9.8 (8.0, 11.8)	<0.001
IgA	0.44 (0.24, 0.73)	1.20 (0.83, 1.68)	<0.001
LDH	356 (302, 427)	307 (263, 366)	<0.001
*β*2 microglobulin	2.79 (2.34, 3.37)	2.06 (1.75, 2.38)	<0.001
Globulin	24.2 (21.3, 26.4)	28.3 (26.0, 30.5)	<0.001
AGR	1.80 (1.60, 2.10)	1.50 (1.30, 1.60)	<0.001
D-dimer	0.43 (0.28, 0.76)	0.47 (0.33, 0.69)	0.14
Sex			0.012
Female	132 (39%)	339 (47%)	
Male	209 (61%)	383 (53%)	
Hospital stay	6 (5, 7)	6 (4, 7)	0.11
Days of fever	3.50 (2.00, 6.00)	3.50 (2.00, 6.00)	>0.9
Cough duration	4 (2, 5)	4 (2, 5)	0.6
Age	3.00 (1.00, 5.00)	3.00 (1.00, 6.00)	0.2

a Median (Q1, Q3); *n* (%).

b Wilcoxon rank sum test; Pearson's Chi-squared test.

### Feature selection

The initial dataset comprised 20 potential predictor variables, including demographic characteristics, clinical presentation, laboratory findings, and radiological features from 1,063 pediatric patients with confirmed CAP. To address high dimensionality and identify the most relevant predictors, a two-stage feature selection process was implemented. First, we selected the 13 biomarkers with significant differences (Wilcoxon rank sum test, *P* < 0.01) between the HRSV and MP groups (eosinophilic granulocyte, CK, creatine kinase isoenzyme MB, immunoglobulin M/G/A, LDH, CRP, globulin, *β*2-microglobulin, AGR, procalcitonin, and glutamic-pyruvic transaminase).

**Figure 1:**
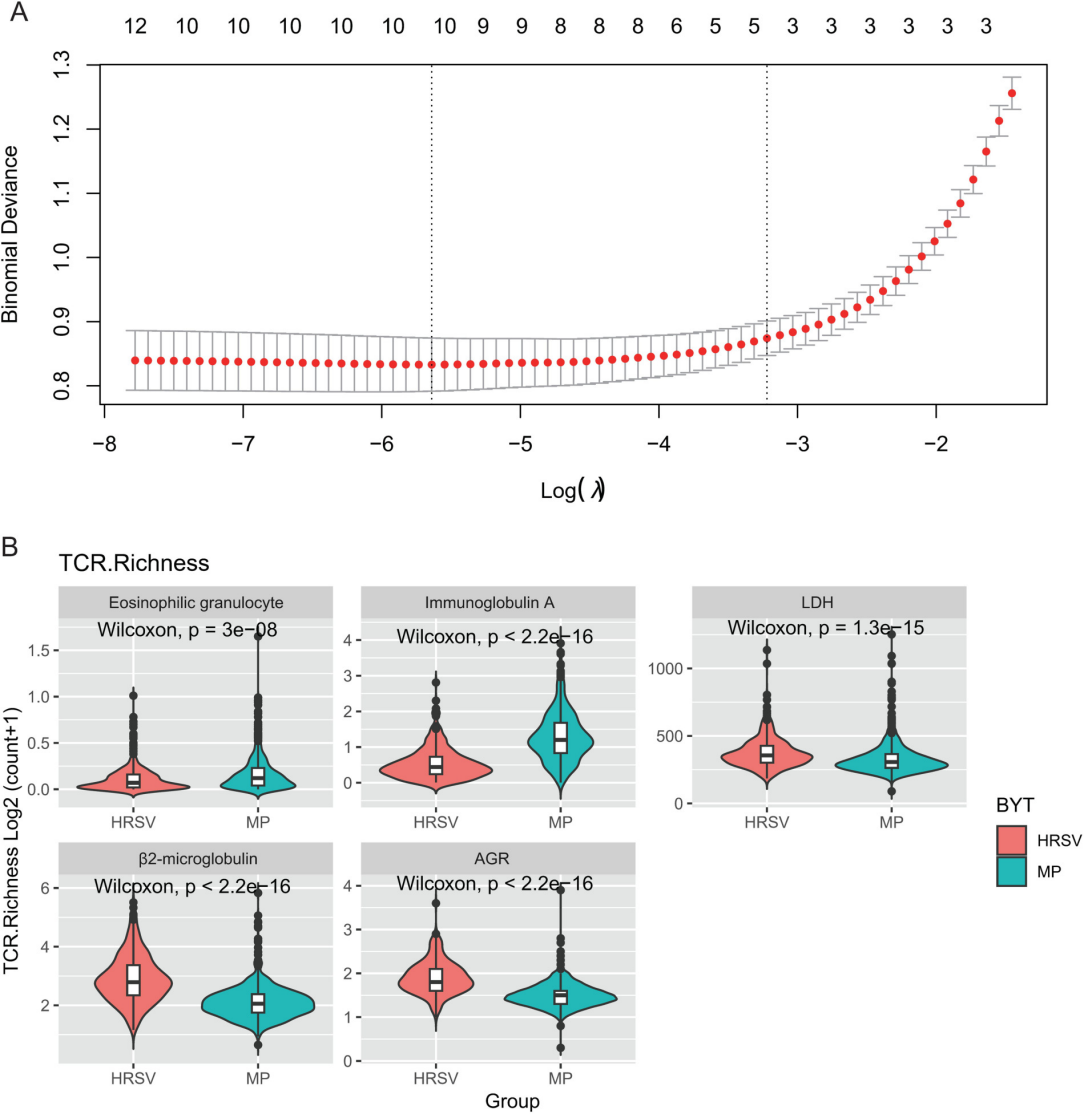
Feature selection for discriminating pediatric HRSV from MP infection. **(A)** Feature selection integrated by LASSO. The left vertical dotted line shows where the CV-error curve hit its minimum (lambda.min) and the right vertical dotted line shows the most regularized model with a CV-error within 1 standard deviation of the minimum (lambda.1se). **(B)** The violin plot shows the differences in LASSO-derived variables between the two groups caused by HRSV and MP infection. The median is indicated with a transverse line in the interior of the box.

Subsequently, to further refine the feature set and confirm the importance of the pre-selected variables, the LASSO regression was applied within a resampling (10-fold cross-validation) framework to shrink the coefficients of non-informative variables to zero, effectively performing feature selection. The optimal lambda value was selected based on the minimum binomial deviance rule from the cross-validation. LASSO regression analysis was applied to the 13 preselected variables and determined the number of variables varies between 5 (1 se, coefficient: 0.03992896) and 10 (min, coefficient: 0.003554536, Figure 1A). Given a larger number of variables are included under the minvalue, which can increase the accuracy of the model but may prone to overfitting, we used 1 se incorporated into the model construction. This hybrid selection process culminated in a parsimonious yet highly informative set of five predictors for the final model: eosinophilic granulocyte, IgA, LDH, *β*2-microglobulin, and AGR (Figure 1B).

### Model development and performance

**Figure 2:**
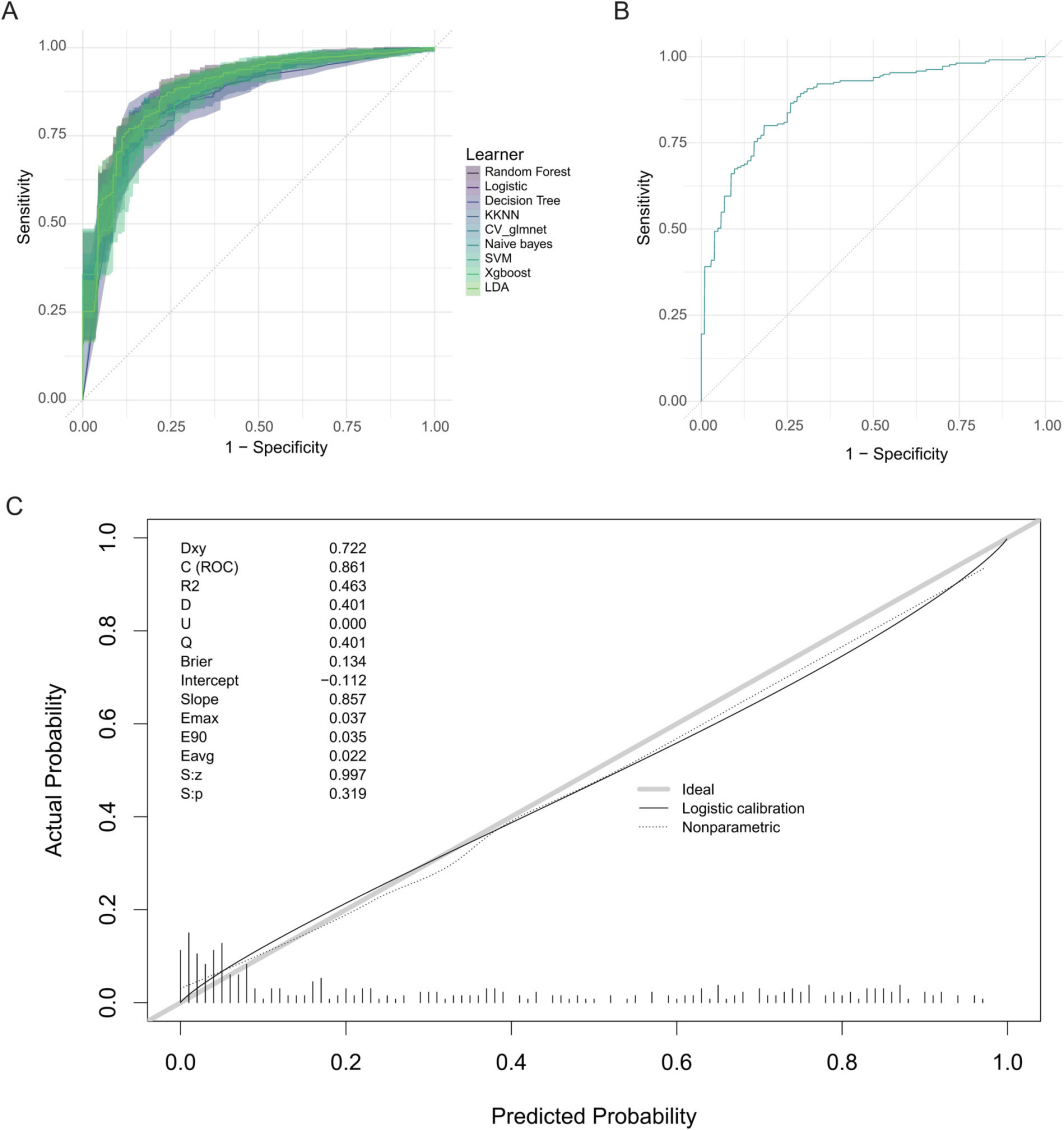
ML performance by hybrid algorithms. **(A)** Model performance of discriminating pediatric HRSV from MP infection. ROC curves for each model in the YMCHH training cohort. The RF model demonstrated the best predictive performance. **(B)** ROC curve for RF model in the YMCHH test set. **(C)** A calibration plot for the internal YMCHH test set.

Prior to model building, the dataset was partitioned into a training set (*n* = 744, 70%) and a hold-out test set (*n* = 319, 30%) to ensure robust external validation. A predictive model for distinguishing HRSV from MP infection was developed using a panel of five blood-based biomarkers: eosinophilic granulocyte, IgA, LDH, β2-microglobulin, and AGR. The RF model demonstrated the optimal discriminatory power (Figure 2A). In the training set, the AUC was 0.89 (95% CI: 0.85–0.90), sensitivity 0.89, and specificity 0.72. The overall accuracy was 83.9%. This high performance was maintained in the internal test set, with an AUC of 0.86 (Figure 2B). In addition, a calibration plot further confirmed the robustness of the model, with a Brier score of 0.134 and the slope of 0.857 in the internal test set (Figure 2C).

We further calculated the feature importance within the model and found that the top three contributors to the prediction were: IgA (score: 80.82), β2 microglobulin (72.89), and AGR (52.98).

### Comparison with single biomarker

The composite biomarker model was significantly superior to any single biomarker for differential diagnosis. The AUC of the model (0.89) was higher than the AUCs for eosinophilic granulocyte alone (0.55), IgA alone (0.78), β2-microglobulinalone (0.74), AGR alone (0.83), and LDH alone (0.63).

## Discussion

The development of a blood signature for distinguishing pediatric HRSV from MP pneumonia represents a significant advancement in the quest for rapid, non-invasive, and accurate diagnostic tools for childhood respiratory infections. Our study successfully constructed and validated a predictive model using a panel of peripheral blood parameters, demonstrating high discriminatory power. Nevertheless, what we need to be cautious about is that this model has a reduced ability to distinguish between cases of combined bacterial and viral infections (AUC = 0.76), suggests that in the future, a new model specifically tailored for combined infections will need to be flexibly re-constructed. The core challenge in pediatric pneumonia management lies in the clinical overlap between viral and bacterial etiologies, which can lead to inappropriate antibiotic use and delayed targeted care. Our model directly addresses this by transforming accessible blood test results into a probabilistic etiological signature, thereby facilitating more precise clinical decision-making at the point of care.

The superior performance of our model can be attributed to its foundation in the distinct host immune responses elicited by HRSV and MP. The feature importance analysis within our model highlighted parameters related to eosinophilic granulocyte dynamics, immune factors, and specific cytokine levels as key discriminators. This finding resonates with studies in other inflammatory conditions where immune cell ratios and cytokine profiles have proven to be powerful diagnostic markers. For instance, in acute graft-vs.-host disease, cytokine signatures were found to outperform immune subset data alone in ML models (18). Similarly, in Kawasaki disease, a pediatric vasculitis, platelet-associated biomarkers and immune dysregulation profiles were successfully harnessed to build diagnostic models (19). Our work extends this paradigm to the etiological differentiation of common pediatric pneumonias, confirming that the systemic immune “fingerprint” detectable in peripheral blood holds substantial diagnostic information.

When compared to existing diagnostic methods, our blood-based signature offers several potential advantages. Traditional methods rely on pathogen detection (e.g., PCR and culture), which can be time-consuming, expensive, and sometimes insensitive, especially after antibiotic initiation or due to sampling issues (20). It should be noted that some markers such as LDH and β2-microglobulinin the model may be not routinely detected in some hospitals with limited conditions, alternatively integrating granulocyte, IgA, and AGR also presented a good predictive power (AUC > 0.8) but warrants further external validation. In pediatric sepsis, a nomogram combining serum HMGB1 with PCT showed excellent prognostic accuracy for septic shock, highlighting the power of combining biomarkers (21). Our model operates on a similar principle but is tailored for etiological differentiation at an earlier diagnostic stage.

However, our study has some limitations, which must be openly acknowledged to guide future research. The retrospective, single-center design is a primary constraint, potentially introducing selection bias and limiting the generalizability of our findings. While internal validation showed robust performance, external validation in independent, multi-center, and prospective cohorts is essential. The blood parameters we used, may not capture the full spectrum of the host response. The potential contribution of cellular immune factors to disease differentiation also cannot be ruled out since no cellular immune indicators were included in statistical analysis. Furthermore, the model currently provides an etiological probability but does not directly predict disease severity or complications. Future iterations could integrate clinical data (e.g., oxygen saturation, imaging findings) to create a comprehensive risk stratification tool.

In conclusion, we have demonstrated the feasibility and high accuracy of a ML-based blood signature for differentiating pediatric HRSV from MP pneumonia. The biological plausibility of the signature, rooted in divergent immune pathways, strengthens its validity. Future research must focus on rigorous external validation and technological integration into clinical workflows, leading to more targeted therapies, improved outcomes, and wiser stewardship of antimicrobial agents.

## Data Availability

The original contributions presented in the study are included in the article/Supplementary Material, further inquiries can be directed to the corresponding author/s.
