# Supplementary figures and images for: Machine learning based development of an early diagnosis signature for distinguishing hospitalized pediatric human respiratory syncytial virus infection from mycoplasma pneumonia
Source: Front Pediatr. 2026 Jun 2;14:1845227. doi: 10.3389/fped.2026.1845227 (PMC13269324; doi:10.3389/fped.2026.1845227)

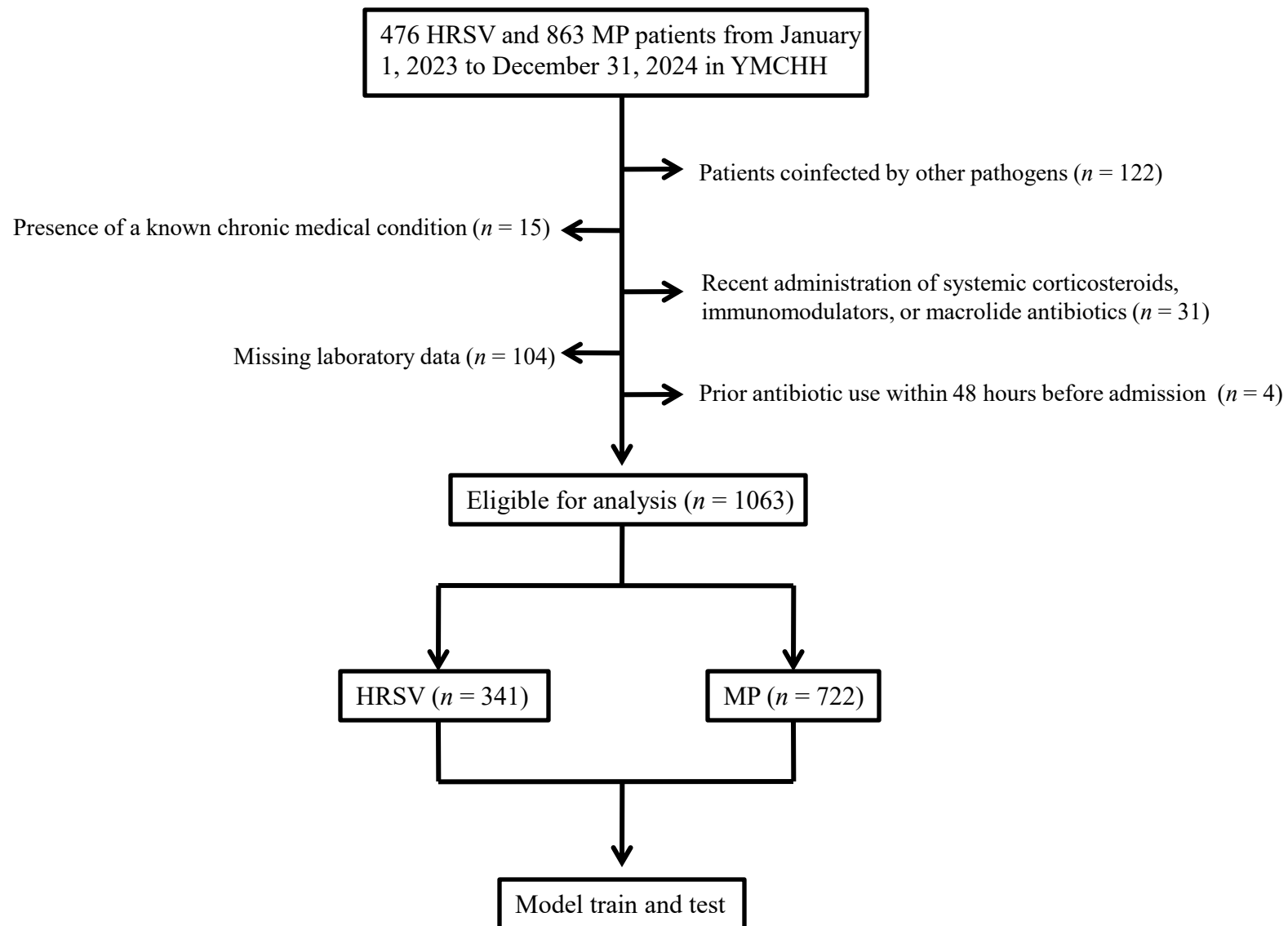

Figure S1. Flowchart for patient inclusion/exclusion and analysis in this study.

Supplement: Supplementary file 1 [file Datasheet1.pdf]
